# Transformative Potential of AI in Healthcare: Definitions, Applications, and Navigating the Ethical Landscape and Public Perspectives

**DOI:** 10.3390/healthcare12020125

**Published:** 2024-01-05

**Authors:** Molly Bekbolatova, Jonathan Mayer, Chi Wei Ong, Milan Toma

**Affiliations:** 1 Department of Osteopathic Manipulative Medicine, College of Osteopathic Medicine, New York Institute of Technology, Old Westbury, NY 11568, USA; mbekbola@nyit.edu (M.B.); jmayer03@nyit.edu (J.M.); 2 School of Chemistry, Chemical Engineering, and Biotechnology, Nanyang Technological University, 62 Nanyang Drive, Singapore 637459, Singapore

**Keywords:** predictive modeling, artificial intelligence, medicine, computational models, forecasting, future

## Abstract

Artificial intelligence (AI) has emerged as a crucial tool in healthcare with the primary aim of improving patient outcomes and optimizing healthcare delivery. By harnessing machine learning algorithms, natural language processing, and computer vision, AI enables the analysis of complex medical data. The integration of AI into healthcare systems aims to support clinicians, personalize patient care, and enhance population health, all while addressing the challenges posed by rising costs and limited resources. As a subdivision of computer science, AI focuses on the development of advanced algorithms capable of performing complex tasks that were once reliant on human intelligence. The ultimate goal is to achieve human-level performance with improved efficiency and accuracy in problem-solving and task execution, thereby reducing the need for human intervention. Various industries, including engineering, media/entertainment, finance, and education, have already reaped significant benefits by incorporating AI systems into their operations. Notably, the healthcare sector has witnessed rapid growth in the utilization of AI technology. Nevertheless, there remains untapped potential for AI to truly revolutionize the industry. It is important to note that despite concerns about job displacement, AI in healthcare should not be viewed as a threat to human workers. Instead, AI systems are designed to augment and support healthcare professionals, freeing up their time to focus on more complex and critical tasks. By automating routine and repetitive tasks, AI can alleviate the burden on healthcare professionals, allowing them to dedicate more attention to patient care and meaningful interactions. However, legal and ethical challenges must be addressed when embracing AI technology in medicine, alongside comprehensive public education to ensure widespread acceptance.

## 1. Introduction

The origins of AI can be traced back to more than 70 years ago, specifically during World War II. It was during this time that mathematician Alan Turing developed The Bombe, an electro-mechanical computer spanning almost 50 square feet in size. A remarkable feat at the time, The Bombe successfully broke the Enigma code, a task previously believed to be impossible even for the most brilliant human mathematicians [1]. This pivotal moment in history ignited further inquiry into the potential of creating machines capable of incorporating external data and processing it using algorithms to produce useful and efficient outcomes for various tasks. In 1950, Alan Turing examined the possibility of creating intelligent computational machines that could imitate human thought processes. He developed a method known as the Turing test to assess whether a machine’s responses can be distinguished from those of a human [2]. If the machine’s answers are indistinguishable from a human’s, it is considered intelligent. Several years later in 1956, John McCarthy coined the term “Artificial Intelligence”, describing it as the computational aspect of achieving goals and capable of being precisely defined for simulation by machines [3].

Since the inception of AI, this field has experienced rapid advancement. Following its successful demonstration in passing the Turing test, AI began to make significant contributions across various industries. One early adopter was the automotive industry, which witnessed an infusion of AI technology starting in the early 1960s. In particular, John Devol and Joseph Engelberger pioneered the development of Unimate, a groundbreaking industrial robot introduced in 1961. Originally designed for die-casting tasks, it later found application in workpiece handling and spot-welding processes involved in car body manufacturing [4]. Subsequently, advancements were made towards creating conversational systems using chatbots powered by AI algorithms. In 1966, Joseph Weizenbaum introduced Eliza, the first chatbot. Eliza could engage in conversations with humans by utilizing pattern matching to generate appropriate responses based on textual inputs [5]. During the same period, Charles A. Rosen created Shakey, a pioneering achievement that enabled an electronic entity capable of executing complex tasks and identifying errors for correction [6]. These significant milestones played a crucial role in shaping AI’s impact across industries and its subsequent expansion into healthcare applications.

**Figure 1:**
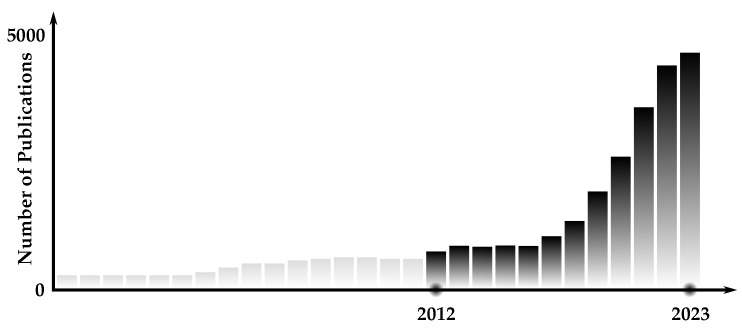
Number of PubMed indexed publications on AI in medicine.

During the period from the 1970s to the 2000s, there was a decline in significant advancements in AI known as the “AI winter”. However, this downturn resulted in increased utilization of AI in the medical field [7]. Ophthalmology emerged as one of the early adopters of incorporating AI technology. A notable example was the development of CASNET/glaucoma model in 1976, which utilized stored patient data to make informed decisions based on physiological parameter changes, clinical manifestations, and treatment outcomes [8]. This model showcased the potential of AI in ophthalmology and paved the way for further advancements in the field. A similar system known as the INTERNIST-1 was developed to assist physicians in accurately diagnosing complex internal medicine diseases [9]. In 1996, the ZEUS robotic surgery system was introduced as the first complete remote surgery system, designed to enhance laparoscopic procedures by eliminating motion scaling and tremors [10]. These early advancements in AI within the medical field have sparked renewed interest in AI applications throughout medicine in the 21st century. Figure 1 illustrates the significant surge in AI research over the past decade.

In recent years, there has been a significant surge of interest in integrating AI into the medical domain. Continuous advancements and enhancements of AI have paved the way for its application in complex medical cases. For instance, in 2017 AI technology was introduced to cardiology through CardioAI, an innovative clinical design that utilizes deep learning algorithms to analyze cardiac magnetic resonance images rapidly. This groundbreaking development enables the swift determination of essential information such as cardiac ejection fraction within seconds [11]. Subsequently, similar techniques were extended to lung imaging analysis and interpretation of musculoskeletal system X-ray images as well as head CT scans. A noteworthy step forward occurred in 2019, when AI was utilized collaboratively with endoscopies, demonstrating promising potential for future growth while remaining subject to ongoing refinement and improvement. The use of computer-aided diagnosis (CAD) has enabled accurate detection, differentiation, and characterization of both neoplastic and non-neoplastic colon polyps, with an average accuracy rate of 91.5% [11]. Moreover, the field of AI in healthcare has witnessed the emergence of several companies that contribute to various aspects of medicine; for examples, see Table 1. Such companies support robotic surgeries, data collection from patients, and prompt diagnosis and treatment through blood tests for early-stage cancers. This highlights how AI has evolved into an effective tool from its inception in the 1950s and continues to hold potential for further advancements in addressing challenging tasks encountered within the medical domain.

This review contributes to the field by: (a) providing readers with a clear and concise understanding of the topic through a detailed overview of AI, including its origins, evolution, and current state, with a particular focus on its uses within healthcare; (b) reviewing and analyzing varied applications of AI in healthcare, ranging from diagnostics and medical imaging to surgical assistance, patient prognosis, and treatment decision-making; (c) discussing AI’s role and impact during pandemic scenarios, demonstrating its potential in disease identification, monitoring patients, determining mortality risk, managing data, and facilitating rapid response, accurate diagnosis, and efficient treatment; (d) highlighting the potential advantages and challenges associated with the integration of AI in healthcare, including ethical, legal, and social considerations, while raising awareness about the need for regulated and ethically guided use of AI in medicine; (e) detailing the role of AI in contemporary innovations such as remote monitoring, telehealth, drug discovery, vaccine development, and natural language processing; and (f) providing a thorough view of AI’s transformative potential in various medical fields, such as rehabilitation, gastroenterology, nutritional assessment, and surgery.

This paper is organized into several cohesive sections, each serving a specific purpose. (1) Introduction—introduces the topic at hand, establishes its relevance, and presents an overview of the current state of knowledge in the field. It also outlines the objectives of the paper. (2) Key principles of AI—describes the structured methodologies and advanced techniques used in AI, such as artificial neural networks and deep learning, and their successful applications in predictive modeling within the medical domain. (3) Key Applications of AI in Healthcare—explores the diverse applications of AI in health care, ranging from diagnostics and medical imaging to surgical assistance, patient prognosis, and treatment decision-making. (4) The Role of AI in Medicine—delves into the transformative potential of AI in various medical fields, touching upon areas such as rehabilitation, gastroenterology, nutritional assessment, and surgery, among others. (5) Considerations of AI Applications—tackles the crucial ethical, legal, and social considerations that arise from the integration of AI in healthcare, addressing issues related to accountability, liability, privacy protection, bias and discrimination, transparency, consent, and the balance between innovation and regulatory oversight. (6) Discussion—synthesizes the contents of the previous sections, contemplating the implications of the findings, examining potential challenges, and offering thoughts on future directions in the field of AI in healthcare. This structure ensures comprehensive coverage of the topic and aids readers in understanding the various aspects of the AI in healthcare.

## 2. Key Principles of AI

**Figure 2:**
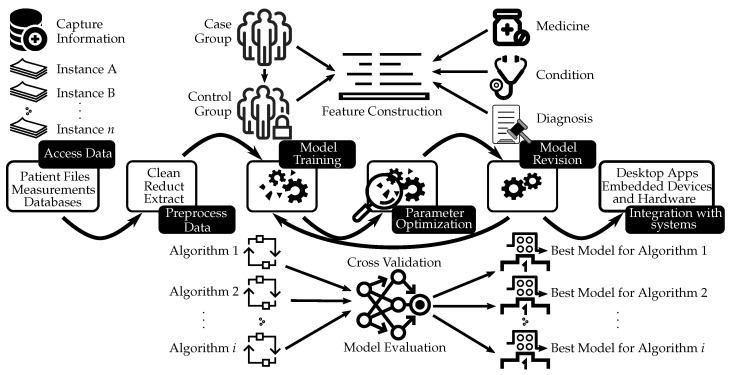
The use of neural networks for medical decision support. This procedure typically includes multiple stages such as gathering and preprocessing data, developing the model, refining the model via cross-validation, selecting the optimal model, and amalgamating it with systems like desktop software or embedded hardware and devices.

The field of AI utilizes a structured method to integrate external data, employing intricate algorithms that ultimately yield accurate predictions for specific scenarios [12]. This systematic process involves advanced techniques such as artificial neural networks and deep learning. By appropriately selecting and implementing the algorithm tailored to the technique being employed [13], AI has successfully found its applications in predictive modeling within the medical domain. Figure 2 depicts the use of neural networks for medical decision support. This process might encompass various steps such as collecting and preprocessing data, training the model, revising the model through cross-validation, choosing the best model, and integrating it with systems such as desktop applications or embedded devices and hardware.

### 2.1. Artificial Neural Networks

**Figure 3:**
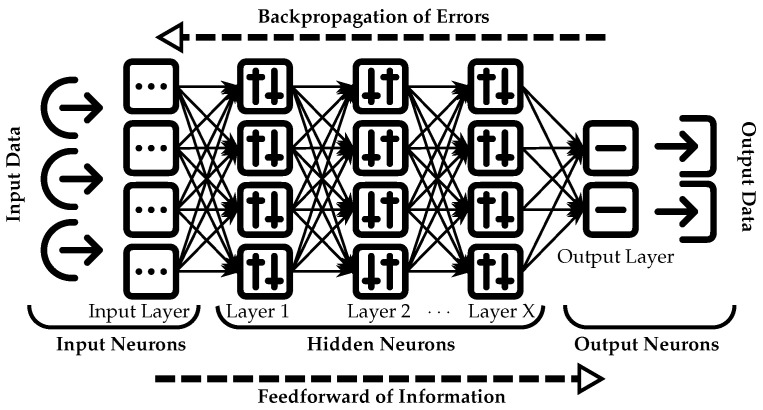
Neural elements of a multilayer feedforward backpropagation network.

Artificial Neural Networks (ANNs) have gained popularity in the medical domain due to their effectiveness in diagnostics and imaging detection. They are commonly used for clinical diagnosis, including histopathology evaluations and electrocardiograms (ECGs). In addition, ANNs have been integrated into surgical diagnostic procedures covering aspects such as abdominal pain assessment, appendicitis identification, detection of retained common bile duct stones, and evaluation of glaucomas [14]. These networks draw inspiration from the nervous system and employ a sophisticated methodology for gathering input data that ensures accurate results are obtained. In the medical field, accurate diagnosis is crucial for effective treatment and patient care. As depicted in Figure 3, artificial neural networks comprise interconnected layers that guide input data towards the intended path in order to produce a reliable output. The input data are initially processed and gathered in the input layer, which captures general information. The data then traverse multiple subsequent layers (layer 1, layer 2, etc.), ensuring greater accuracy of the resulting output. Upon traversing these deeper layers, the data reach the final output layer, where an appropriate solution or answer is generated based on the provided input. ANNs effectively select optimal paths for data while minimizing inaccuracies by eliminating alternative pathways during processing. The application of ANNs in medical diagnosis is becoming increasingly prevalent, as they offer a promising solution for accurate and efficient diagnostics.

In the context of ANNs, “choosing the best path for the data” refers to the network’s ability to evaluate input data and make informed decisions regarding which connections and pathways within the network should be activated. This process involves analyzing patterns and features in order to adjust connection strengths between neurons while giving priority to relevant information. By selecting an appropriate pathway through the network, the probability of producing accurate outputs is increased. To determine this optimal path, various factors are considered by the ANN, including the weights assigned to connections and the activation functions applied to the neurons. These considerations enable informed decision-making with the goal of minimizing errors or inaccuracies in the findings [15].

The “feed forward” model described here relies on a substantial amount of data to effectively determine the optimal pathway. Furthermore, this network commonly utilizes a backpropagation algorithm, in which information flows backwards from the deepest layer to match inputs and outputs by minimizing discrepancies between predicted values and known correct outputs [16]. In essence, the available data are meticulously analyzed to provide an accurate solution.

### 2.2. Deep Learning Networks

Deep Learning Networks (DLNs) are a subfield of AI with a broad scope, encompassing image diagnostics. DLNs are frequently combined with other networks in the same category, including Artificial Neural Networks and Convolutional Neural Networks (CNNs). CNNs specifically fall under the category of ANNs, and adopt a similar methodology. Inspired by the visual perception abilities of living organisms, CNNs consist of multiple layers and employ backpropagation algorithms to directly recognize visual patterns without extensive preprocessing [17]. DLNs share this cascading approach across several layers for information extraction; however, they incorporate additional learning modalities such as unsupervised pattern analysis and supervised classification into their framework as well. Supervised learning focuses on establishing a connection between input and desired output by utilizing the provided inputs and their corresponding correct outputs. The algorithm learns by comparing its actual output to the correct outputs, enabling it to identify errors and making it beneficial for tasks such as classification and prediction [18]. In contrast, unsupervised learning involves finding solutions to unlabeled information without any given guidance. The algorithm must explore the provided data in order to create a meaningful structure. This process relies on hierarchical representation, in which higher-level features are derived from lower-level ones, resulting in a hierarchy of concepts being formed [18]. Overall, deep neural networks play an integral role in incorporating neural networks alongside various learning modalities for efficient analysis of data and representation of images. Therefore, DLNs, particularly CNNs, are widely used in image diagnostics thanks to their ability to directly recognize visual patterns without extensive preprocessing.

### 2.3. Machine Learning

Machine Learning (ML) is an extensive branch of AI that encompasses various types of networks. It shares similarities with DLNs in terms of utilizing supervised and unsupervised learning techniques. Additionally, ML incorporates a third learning modality called semi-supervised learning, which combines aspects of both supervised and unsupervised approaches. The primary objective of this approach is to leverage unlabeled data as a way to enhance model performance [19]. ML is a distinctive branch of AI that utilizes datasets to learn and draw correlations between variables [20]. Unlike traditional problem solving techniques, ML does not rely on predefined step-by-step processes. Instead, it leverages the power of data analysis to identify patterns and trends in specific characteristics for accomplishing complex tasks [21]. This integrative approach incorporating various subfields makes ML an invaluable tool applicable across diverse industries.

### 2.4. Key Principles of AI within the Context of Healthcare

To reiterate the above within the context of healthcare, the relationships between ANNs, DLNs, and ML are described below to provide a more comprehensive understanding of their interplay within the field of AI. ANNs play a fundamental role in AI for healthcare applications. ANNs are computational models inspired by the structure and functioning of biological neural networks. They have been extensively utilized in various healthcare applications, including disease diagnosis, prognosis, and treatment prediction. They consist of interconnected artificial neurons that process and transmit information in a manner similar to the human brain. ANNs have demonstrated remarkable capabilities in pattern recognition, feature extraction, and nonlinear mapping, making them highly effective in analyzing complex medical data. DLNs build upon the foundations of ANNs, and have revolutionized the field of AI in healthcare. DLNs are composed of multiple layers of artificial neurons, enabling the creation of deep neural networks capable of learning hierarchical representations of data. This depth allows DLNs to automatically extract complex and abstract features from medical images, signals, and textual data. Their ability to learn from large-scale datasets has facilitated significant advancements in medical imaging analysis, natural language processing, and clinical decision support systems.

The relationship between ANNs and DLNs is progressive, with DLNs representing a more advanced and complex form of neural network architecture. While ANNs laid the groundwork for neural network-based approaches, DLNs have extended these capabilities by introducing deeper architectures and more sophisticated learning algorithms. This progression has enabled the development of highly accurate and robust models in healthcare applications. ML encompasses a broader range of techniques including both ANNs and DLNs. It refers to the field of study that focuses on algorithms and models capable of automatically learning from data and making predictions or decisions without being explicitly programmed. ML techniques in healthcare leverage the power of ANN and DLN models, along with other approaches such as decision tree [22], support vector machine [23], and random forest [24]. These techniques enable the extraction of valuable insights from healthcare data, facilitating tasks such as patient risk stratification, treatment recommendation, and health outcome prediction. It is important to note that within the context of this manuscript ML refers to the specific range of techniques employed in the analysis of healthcare data, incorporating both ANN and DLN. This distinction is important to remember in order to avoid any potential confusion regarding the scope of ML in the context of this review.

Additionally, different ML algorithms are particularly suited to different applications. For example, certain models might perform better in image analysis for diagnosis, while others might be more effective in forecasting disease spread. Comparing the efficacy of different ML models using the same datasets is of paramount importance in the field of AI. Such comparisons allow researchers and practitioners to gain insights into the strengths and weaknesses of various models, enabling them to make informed decisions about which approach to adopt for a given task. By applying multiple models to the same dataset, it becomes possible to assess their performance, accuracy, and generalization capabilities, e.g., [25]. This process promotes a better understanding of the underlying algorithms and their suitability for specific problem domains, and can aid in identifying the most effective and efficient solution. Additionally, comparing different models on the same datasets fosters fair and unbiased evaluations, contributing to the advancement of machine learning techniques and driving innovation in the field.

## 3. Key Applications of AI in Healthcare

The various subfields and modalities of AI discussed earlier have the potential to greatly impact different industries, leading to overall improvements and growth. The recent COVID-19 pandemic has created a pressing need for digital solutions, particularly in the medical field. Machine learning has found applications in tasks such as medical imaging, disease diagnosis, patient prognosis, and treatment decision-making [26,27,28]. Moreover, AI has been successfully integrated into other important aspects of healthcare such as virtual nursing assistants and reducing medication errors [29]. As shown in Table 2, the majority of AI applications are within the medical field due to several key factors that must be addressed for optimal patient care outcomes. Notably, these advancements hold significant financial value, with an estimated combined annual worth exceeding USD 100 billion over the next three years.

The key applications of AI in healthcare include the following. (a) Medical Imaging and Radiology: AI is used in diagnosing diseases and conditions through the analysis of images such as X-rays, CT scans, and MRIs [30]. (b) Clinical Trial and Research: AI can streamline the process of clinical trials and research by improving patient selection, monitoring, and data collection [31]. (c) Managing Electronic Health Records (EHRs): AI can enhance the management of EHRs, including information capturing, data analysis, and dealing with natural language processing [32]. (d) Diagnosis and Clinical Decision Making: AI algorithms significantly contribute to the diagnosis of various diseases, including cancer [33], and play a crucial role in clinical decision-making [34]. (e) Surgeries: AI-powered robots such as the Da Vinci surgical system and the Smart Tissue Autonomous Robot can perform surgeries with greater precision and accuracy [35]. (f) Personalized Medicine: AI may help tailor therapies based on individual genetic information and patient-specific data [36], improving the success rates in cancer treatments, for instance [37]. (g) Risk Assessment and Prognostication: AI can process historical and real-time data to identify high-risk patients, predict disease progression, and inform clinical decision-making [38,39,40]. (h) Telemedicine and Virtual Health Assistants: AI can provide assessments and basic healthcare recommendations, improving access to healthcare services [41,42]. (i) Drug Discovery and Development: AI fast-tracks the drug discovery process and can establish effective drug combinations for complex diseases such as cancer [43]. (j) Patient Monitoring and Care: AI can monitor patient symptoms in real time, supporting healthcare providers in promptly detecting and addressing complications [44]. These applications highlight the potential of AI to significantly enhance the efficiency, accuracy, and precision of healthcare delivery from diagnosis to treatment and follow-up care.

## 4. The Role of AI in Medicine

In order to fully comprehend the extent of AI’s contribution to the healthcare industry, an examination of the existing state of medical care is necessary. This includes acknowledging any limitations that can potentially be addressed and resolved through the integration of AI. For example, when patients lack sufficient understanding of medical procedures it hinders their ability to provide informed consent. This inadequacy is mainly attributed to time constraints and a lack of personalization. To address this issue, the utilization of an AI-powered chatbot has been found to improve the informed consent process. In a study, patients were randomly assigned to either a conventional informed consent group or an AI-supported chatbot group [45]. The findings indicated that although satisfaction levels were comparable between the two groups, the AI group exhibited significantly higher levels of accurate comprehension regarding the procedure and its associated risks. The subsequent sections present a comprehensive outline of current challenges within healthcare that stand to benefit from advancements in AI technology.

### 4.1. Financial Burdens in Healthcare

Excessive spending poses a significant challenge to the US healthcare system. Previous research indicates that unnecessary costs in the range of USD 760 to 935 billion are incurred annually, which represents almost 25% of the country’s total healthcare budget [46]. This substantial economic burden weighs heavily on the overall US economy and is predominantly due to inefficiencies within the current healthcare system. The integration of AI into this framework has emerged as a potential solution, with projected cost savings of nearly USD 150 billion anticipated by 2026 [7]. These extra funds generated through AI implementation could be directed towards addressing existing healthcare concerns, thereby enhancing patient care quality and improving outcomes for patients overall.

Additionally, the application of AI technologies in healthcare can help ease financial burdens in several ways beyond administrative task automation. (a) Improved Diagnosis and Treatment: AI can help improve the accuracy and speed of diagnosis and treatment, which can result in fewer unnecessary tests and procedures, thereby reducing costs. Additionally, predictive analytics can help prevent disease before it occurs, reducing the need for expensive treatments and hospital stays. (b) Telemedicine: AI can support telemedicine platforms in diagnosing patient symptoms remotely. This reduces costs associated with hospital visits and provides a more affordable and accessible option for patients who may not have easy access to a healthcare facility. (c) Drug Discovery: AI can speed up the drug discovery process, which is typically a lengthy and expensive endeavor. By predicting how different drugs will interact with the body, AI can help identify promising drug candidates more quickly, potentially saving significant research and development costs. (d) Personalized Medicine: AI can analyze individual health data to help healthcare providers tailor treatments specifically to the patient’s genetic and health profile, potentially increasing treatment efficacy and reducing the need for expensive trial-and-error drug prescribing. (e) Resource Allocation: AI can aid in predicting patient inflow in hospitals and clinics. This helps in better allocation and utilization of resources, reducing wastage and cost overruns. (f) Predictive Maintenance of Equipment: AI can predict when healthcare equipment needs maintenance. Predictive maintenance can help to avoid sudden equipment failures and the costs associated with them, allowing for budgeting of equipment replacement or repair. (g) Insurance Fraud Detection: AI can be used to identify false insurance claims, which can result in significant cost savings for healthcare providers and insurance companies.

### 4.2. Redundant Administrative Tasks

The primary reason for excessive healthcare spending is due to significant allocations towards administrative expenses which encompass various redundant tasks such as reviewing patient records, documenting encounters, and managing medical files [47]. Administrative duties are indispensable in the healthcare system; however, they consume a substantial amount of time and effort that could otherwise be dedicated to providing direct patient care. On average, nurses in the United States spend 25% of their working hours on administrative tasks [48]. While it remains crucial for healthcare professionals to attend to these essential responsibilities, there exists an opportunity to enhance efficiency through the utilization of AI. AI has the potential to automate and streamline healthcare administrative systems, reducing the burden on healthcare providers and optimizing processes [49]. In the emerging era, AI has exhibited a remarkable ability to efficiently carry out administrative functions on par with human capabilities [50]. While it cannot completely supplant the indispensable role of humans in healthcare management, AI presents an opportunity to optimize and streamline these operations, thereby assisting providers in their day-to-day administrative responsibilities. By delegating repetitive organizational tasks to automated systems, valuable time can be liberated for meaningful patient-care interactions. Furthermore, AI can improve accuracy and reduce errors in administrative tasks such as reviewing patient records and managing medical files [51].

### 4.3. Provider Burnout

An increasingly prevalent issue in modern healthcare systems is the phenomenon of provider burnout. Studies have found that burnout is a prevalent issue among healthcare professionals, including physicians and other workers in the field [52]. This syndrome can have detrimental effects on mental well-being as well as on the quality and safety of patient care. To mitigate the effects of burnout and improve the overall well-being of healthcare providers, it is crucial to address the contributing factors that lead to burnout, such as excessive workload and burdensome administrative tasks. Provider burnout refers to a chronic stress reaction that arises from emotional exhaustion, mental health decline, and feelings of depersonalization experienced by healthcare professionals due to systemic and organizational factors [53]. According to recent research findings, approximately 25.6% of physicians reported experiencing symptoms associated with burnout [54]. Interestingly, it was discovered that EHR systems played a significant role in contributing to these symptoms, as indicated by 74.5% of survey respondents attributing EHR as the primary cause [54]. Recognizing the critical impact on patient well-being, it becomes essential to acknowledge the importance of maintaining good physical and mental health among healthcare providers who must perform at their utmost capacity to ensure high-quality care delivery. The issue of burnout among healthcare providers remains a persistent challenge for healthcare organizations globally. Efforts are currently underway to address this issue, including the introduction of natural language processing (NLP)-based AI systems [55]. These NLP analytics can generate insights and analyze patient data on a large scale, offering opportunities to optimize electronic health record systems cost-effectively and efficiently [56]. While integrating NLP in EHR requires continued effort and improvement, the potential benefits of increased efficiency and decreased provider burnout serve as strong motivation for further advancements in this area.

### 4.4. Diagnosis and Clinical Decision Making

The application of AI in the field of medical diagnosis and treatment is not a new concept. Researchers have been exploring whether AI can accurately suggest clinical diagnoses and recommend optimal treatment plans since the 1970s. The ability to improve the accuracy of clinical diagnoses is seen as an appealing aspect of AI in medicine, as early and precise detection of medical conditions may lead to better patient outcomes by reducing readmission rates, preventing progression to chronic conditions, and improving prognosis with a lower risk for complications. Currently, various subspecialties are showing growing interest in developing advanced AI tools that could assist with clinical problem-solving. These tools can analyze large amounts of medical data, including patient records [57], lab results, and imaging data, to identify patterns and correlations that might not be immediately apparent to human clinicians. Thus, the use of AI in clinical decision-making is based on its ability to analyze a vast amount of patient data and identify patterns that can accurately predict clinical outcomes and recommend appropriate treatments. AI algorithms are capable of examining various types of medical information, including radiographic images, laboratory results, and patient records, to assist healthcare professionals in recognizing abnormal patterns indicative of specific diagnoses. Machine learning, an integral component of AI, plays a crucial role in clinical decision-making by analyzing data to make informed decisions and predictions [58]. Deep learning (DL), a subfield within ML, has demonstrated considerable success in diagnosing heart failure (HF). In light of the significant global burden posed by HF, which affects approximately 26 million individuals worldwide, the achievements of DL hold great importance in the healthcare context [58,59].

The integration of ML in the field of orthopedics has shown promising outcomes, as AI can provide valuable assistance to orthopedic surgeons by analyzing radiographic images, conducting preoperative risk assessments, and aiding in clinical decision-making [60]. Particularly for joint arthroplasty, which is a common elective procedure in orthopedics, AI offers significant advantages over traditional methods. By utilizing advanced vision models, sensors, and feedback mechanisms during robot-assisted arthroplasty surgery with augmented intelligence capabilities, AI technology demonstrates superior performance in achieving precise joint alignment and accurate implant placement tailored to individual patients’ needs. This patient-centered approach sets AI-driven augmentation apart from conventional techniques and establishes it as the future direction for arthroplasty procedures [61].

An interdisciplinary application of ML lies in cancer diagnostics, risk stratification, and prognosis [62,63]. These aspects play a crucial role in comprehending the progression of diseases and determining survival probabilities. In the past, medical professionals heavily relied on their expertise acquired through years of clinical practice to guide the diagnosis and treatment of cancer. However, traditional methods have shown limitations such as clinical errors and higher rates of misdiagnosis. By incorporating ML algorithms into oncology practices, there is potential to mitigate these challenges, as AI algorithms have demonstrated superiority in early cancer detection compared to conventional approaches [64,65]. Furthermore, the integration of AI in oncology has shown promising prospects for enhancing success rates in cancer treatments by tailoring therapies based on individual genetic information and patient-specific data [66].

AI algorithms have the ability to conduct clinical screenings for disease symptoms, allowing for an assessment of the probability prior to a diagnosis being made by a healthcare professional. One notable example of this is seen in the identification of Diabetic Retinopathy (DR), where several AI screening tools have been created and are now available for use in certain countries. In the past, identifying DR relied solely on human clinicians or graders [67]. However, deep learning screening tools can accurately classify individuals with DR at a statistically significant rate of 96.8% [68]. By making these automated screening tools accessible to the public, it becomes possible to initiate an appropriate follow-up plan with a clinician much more quickly, thereby reducing the chances of complications resulting from undiagnosed DR.

Numerous endeavors in the past and present have demonstrated AI’s capacity to excel in clinical decision-making across various healthcare domains. In these instances, AI has successfully matched the vast knowledge and expertise of healthcare practitioners. However, it is crucial to recognize that the application of AI as both a diagnostic and treatment tool remains in its experimental stages and is not yet widely accessible for use within clinical settings. The widespread integration of AI into healthcare systems remains an additional hurdle on the path toward achieving full automation. The widespread integration of AI in clinical decision-making is expected to occur within the next 5–10 years. However, this timeline relies on obtaining approval from governing stakeholders, standardizing system operations, educating current professionals, and securing adequate funding [7]. It is important to note that AI currently does not pose an immediate threat to the job security of healthcare professionals, as their expertise remains crucial for providing the human touch in medicine [7]. The development of advanced clinical AI technology aims to enhance physicians’ diagnostic abilities and offer optimal treatment plans rather than replace them entirely.

Physical disabilities are increasingly prevalent with advancing age, and rehabilitation plays a crucial role in restoring function and maintaining independence. However, the limited availability and accessibility of rehabilitation services have hindered their clinical impact. While AI has revolutionized several healthcare domains, its potential in rehabilitation remains uncertain. To address this gap, a systematic review of AI-supported physical rehabilitation technology in the clinical setting was conducted in [69]. The authors’ objectives were to assess the availability of AI-supported physical rehabilitation technology, evaluate its clinical effects, and identify barriers and facilitators to implementation. They identified 28 projects encompassing five categories of AI solutions: app-based systems, robotic devices for function replacement or restoration, gaming systems, and wearables. Among these projects, they analyzed five randomized controlled trials that examined outcomes related to physical function, activity, pain, and health-related quality of life. The results revealed inconsistent clinical effects. Implementation barriers included technology literacy, reliability, and user fatigue, whereas enablers encompassed improved access to rehabilitation programs, remote monitoring of progress, reduced manpower requirements, and lower cost. Hence, while the application of AI in physical rehabilitation is a rapidly growing field, there is a need for more rigorous real-world clinical evaluations and post-implementation experiences in order to fully understand its potential and optimize its benefits. Developers and researchers must strive to conduct robust studies to unlock the true potential of AI in enhancing physical rehabilitation outcomes.

The global prevalence of irritable bowel syndrome (IBS) is approximately 4.1%, leading to decreased quality of life and increased healthcare costs. Current guidelines recommend using symptom-based criteria for diagnosing IBS; however, patients often undergo unnecessary medical interventions. To address this issue, the use of AI in medicine presents a promising solution. This paper aims to review the applications of AI in IBS. AI has proven useful in colonoscopy by detecting organic lesions, diagnosing them, and objectively evaluating the procedure’s quality. Additionally, AI has been used to study biofilm characteristics in the large bowel and establish a potential correlation with IBS [70]. Furthermore, an AI algorithm has been developed to analyze specific bowel sounds associated with IBS. Smartphone applications based on AI have been created to aid in monitoring IBS symptoms. From a therapeutic perspective, an AI system has been designed to recommend personalized diets based on an individual’s microbiota. In conclusion, the future of IBS diagnosis and treatment could greatly benefit from the integration of AI. The era of big data has necessitated the use of AI models to effectively handle the abundance of clinical data available. These data have become invaluable resources for machine learning, with DL models gaining prominence in analyzing unstructured data. However, traditional ML models continue to hold significant potential to enhance healthcare efficiency, especially for structured data. While ML models have been widely applied in predicting diagnoses and prognoses for various diseases, their adoption in gastroenterology has been relatively limited compared to traditional statistical models or DL approaches [71].

The prospect of patients reporting their symptoms to AI-powered NLP systems, followed by receiving diagnostic and treatment assistance from these AI systems, represents a promising avenue in healthcare. With the utilization of NLP, patients would have the capability to articulate their symptoms in a conversational manner, providing a comprehensive account of their medical condition. The AI systems would then employ advanced algorithms to process and interpret the information shared by the patients to integrate it with pertinent medical data. This amalgamation of patient-reported data and contextual knowledge would enable the AI systems to generate insightful analyses, potentially aiding physicians in arriving at accurate diagnoses and formulating tailored treatment plans. The integration of AI and NLP in this context has the potential to enhance patient outcomes by streamlining healthcare delivery and empowering healthcare providers with AI-facilitated decision support tools, thereby fostering more efficient and personalized medical care [72].

The application of AI in healthcare with potential benefits extending to the field of nutrition assessment has garnered significant attention in recent years. Nutrition assessment plays a pivotal role in healthcare, as it provides crucial insights into an individual’s dietary intake, nutritional status, and overall health. Digital technologies such as mobile applications and wearable devices have emerged as valuable tools in facilitating the collection and analysis of dietary data. These technologies enable individuals to track their food consumption, monitor nutritional content, and receive personalized recommendations. AI algorithms enhance the capabilities of these digital tools by utilizing ML and data analytics techniques to process and interpret vast amounts of nutrition-related data. This allows for more accurate and timely assessment of nutritional status, identification of dietary patterns, and evaluation of health risks associated with inadequate or excessive nutrient intake [73].

### 4.5. Surgeries

The expanding application of AI in the healthcare field has brought about significant changes to surgical practices. Surgeons now have access to advanced robotic systems such as Da Vinci that enable less invasive and more independent procedures [74]. Robots are able to perform surgeries on previously inaccessible or delicate areas, such as neural structures, with improved dexterity, speed, and stability. As a result of these advancements, hospital stays have been shortened, recovery times have accelerated, and patient outcomes have been enhanced in terms of morbidity and mortality rates [75]. A recently developed semi-autonomous surgical robot called Smart Tissue Autonomous Robot (STAR) exhibits greater precision and accuracy than experienced surgeons when it comes to incising and suturing soft tissue [76]. Furthermore, studies have indicated that supervised autonomous robot surgery can outperform traditional manual surgery in complex intestinal anastomosis operations, which demand great skillfulness as well as delicacy from surgeons [74,77].

One of the main challenges in traditional surgery is the lack of uniformity caused by variations in surgical experience, training, and skill levels among physicians. However, advancements such as the above-mentioned STAR have addressed this issue by demonstrating the ability to perform surgeries with greater precision and consistency. The STAR system utilizes three-dimensional techniques that result in more consistent suture spacing and improved resection margins compared to manual procedures. Consequently, this reduces the risk of post-surgical complications arising from variability between surgeons’ performances [78]. Moreover, predicting surgical outcomes becomes challenging due to inconsistencies in patient anatomy. Nevertheless, STAR tackles this challenge effectively by adapting to inter-patient anatomical differences and tissue deformability during surgery through real-time data processing and regular updates to the surgical plan [78]. Advancements made on the development of STAR promise not only enhanced surgical outcomes but also increased predictability and standardization within surgeries.

Surgical procedures are widely acknowledged to pose significant risks to the well-being and safety of patients, primarily due to their unpredictable nature and the potential for human error. The integration of AI into surgical practices has been pursued as a means of addressing these concerns and ensuring enhanced levels of safety. Research indicates that robotic surgery boasts a notably lower mortality rate, with 0.097% compared to manual open surgery’s rate of 0.92% per 10,000 surgeries [79]. Thus, by complementing physicians’ expertise with precise robotic assistance, this technology improves surgical accuracy by approximately 6.4% while reducing surgeons’ workload by up to 44% [80].

In addition to its applications in the operating room, AI technology has the potential to enhance healthcare outcomes by streamlining surgical decision-making and management both before and after surgery. As surgery is a significant and often anxiety-provoking event for patients, surgeons need to provide comprehensive support and counseling throughout the entire process. Conversely, surgical interventions necessitate extensive preparation that can be time-consuming; aside from performing procedures, surgeons must analyze various datasets such as patient scans and lab work to develop intricate surgical plans. Fortunately, AI offers a solution by efficiently processing large amounts of patient data within a short timeframe while generating optimal solutions. This allows surgeons more opportunity to engage in meaningful interactions with their patients [81].

A recent literature review analyzed 46 studies on the applications of AI and ML in spinal surgery [82]. The findings revealed that AI/ML models were accurate, with an average overall value of 74.9%. These models performed well in preoperative patient selection, cost prediction, length of stay, functional outcomes, and postoperative mortality prediction. Regression analysis was the most commonly used application, while deep learning/artificial neural networks had the highest sensitivity score of 81.5%. Despite AI/ML’s relatively recent adoption, as shown by 77.5% of studies being published after 2018 (depicted in Figure 1), the results have been encouraging. The increasing prevalence of Big Data and National Registries suggests that the field of spine surgery will gradually adopt and integrate AI/ML into clinical practices, leading to significant improvements in patient care.

### 4.6. Medical Imaging

**Figure 4:**
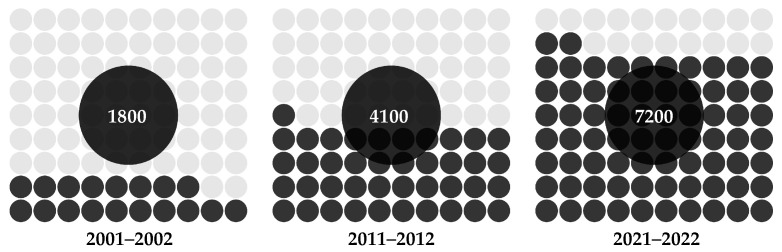
Trends in the number of PubMed-indexed publications on AI in medical imaging.

The introduction of X-ray imaging over 120 years ago revolutionized the way healthcare providers diagnose and treat patients [83]. Since then, advancements in imaging technologies have greatly enhanced the medical field by providing a detailed visualization of vital patient anatomy that is not perceptible to the human eye alone. Despite these innovations, modern imaging technology continues to rely on high-quality images and skilled practitioners to accurately interpret and identify potential ailments [84]. An important development occurred approximately 50 years ago when AI capabilities expanded beyond data analysis and problem-solving to include image recognition and interpretation with a lower error rate compared to radiologists [85,86]. However, it is crucial to dispel public apprehension, as AI does not aim to replace the expertise of radiology professionals with robotic systems; rather, it complements their skills within this discipline. The integration of AI in radiology has gained significant attention, and is expected to enhance the diagnostic process by supplementing radiologists with AI algorithms. This approach aims to streamline operations, reduce redundancy, and improve accuracy in image interpretation [87]. During the COVID-19 pandemic, AI proved invaluable in detecting asymptomatic individuals infected with SARS-CoV-2 on CT scans, allowing radiologists to confidently rule out the disease [88,89]. The use of AI visual recognition tools has played a crucial role in preventing transmission through false negative results. As shown in Figure 4, there has been a noticeable surge of interest among researchers studying the applications of AI technology to medical imaging in recent years [90].

AI plays a vital role in automating echocardiographic analysis, aiding in the diagnosis of cardiovascular diseases. A recent literature review discussed AI algorithms used in various stages of analysis, including image acquisition, view classification, cardiac chamber segmentation, and quantification of cardiac structure and function [91,92]. The authors found that such AI models demonstrate high accuracy in comparison to human experts. The same review explored the potential benefits and limitations of AI in healthcare, such as the need for larger datasets and to address algorithm biases to allow for wider clinical adoption.

Endoscopic ultrasound (EUS) has emerged as a widely utilized diagnostic tool for digestive diseases. With the gradual recognition of AI in healthcare, its superiority in the field of EUS has become increasingly evident. Research findings demonstrate that EUS-AI exhibits superiority, or at least equivalence, to conventional methods of diagnosis, prognosis, and quality control for subepithelial lesions, early esophageal cancer, early gastric cancer, and various pancreatic diseases, including pancreatic cystic lesions, autoimmune pancreatitis, and pancreatic cancer [93]. The implementation of EUS-AI has opened up new avenues for individualized precision medicine while introducing innovative approaches to diagnosing and treating digestive diseases.

### 4.7. AI’s Role in Pandemics

The COVID-19 pandemic has underscored the potential of AI in effectively addressing healthcare challenges. It spurred the adoption and integration of AI technologies into medical practices, showcasing their ability to facilitate rapid response, accurate diagnosis, efficient treatment, and extensive research endeavors [94]. The increased application of AI in healthcare is evident through the noticeable surge in academic publications, as depicted in Figure 1. Importantly, this rise coincided with the onset of the COVID-19 pandemic. Throughout this crisis period, various applications of AI played a crucial role in diverse aspects such as pandemic response management, patient care enhancement, and innovative research initiatives. One of the key areas where AI can be utilized during pandemics is in the management of pandemic responses [95]. AI has proved instrumental in disease cluster identification, monitoring patients, determining mortality risk, disease diagnosis and management, contact tracing through geotagging, resource allocation, and data management. The following sections offer an elaboration on these key areas.

#### 4.7.1. Diagnosis and Screening

In recent years, significant progress has been made in the development of AI-powered diagnostic tools designed to support healthcare professionals in identifying cases of COVID-19 using medical images. These advanced tools utilize convolutional neural networks (CNNs) to carefully examine chest X-rays and CT scans for potential indications of the virus within lung tissue. By analyzing large datasets containing both infected and non-infected images, these AI algorithms are trained to detect subtle patterns that could suggest the presence of COVID-19. This valuable assistance enables radiologists and clinicians to make faster and more precise diagnoses, particularly in regions where PCR testing (i.e., Polymerase Chain Reaction testing, the “gold standard” for COVID-19 tests) may be limited or subject to delays. The ability of AI systems to swiftly process medical images plays a pivotal role in facilitating early detection and treatment plans, ultimately influencing patient outcomes positively and helping to mitigate the spread of the virus [96]. Moreover, AI technology has proven to be beneficial in predicting the progression of COVID-19 cases. By analyzing patterns in symptoms and clinical data, AI algorithms can generate predictive models that help healthcare professionals anticipate how a patient’s condition may develop over time. These predictions guide medical professionals in making informed decisions regarding the appropriate course of treatment and allocation of resources.

#### 4.7.2. Drug Discovery and Vaccine Development

The integration of AI has significantly transformed the field of drug discovery. By utilizing AI algorithms, scientists are now able to simulate and predict the interactions between potential drugs and viral proteins. This is achieved through molecular docking, a technique that assesses how a drug molecule binds to its target protein. In addition to molecular docking, AI-enhanced molecular dynamics simulations provide valuable insights into the behavior of drug–protein complexes over time. These simulations aid researchers in selecting promising candidates for further testing by offering detailed information on their stability and efficacy. Moreover, AI models have been instrumental in vaccine development [97]. Through advanced prediction techniques, these models can accurately define viral protein structures and analyze potential epitopes that can trigger immune responses. By leveraging such predictions, researchers can design vaccines with greater precision that efficiently elicit strong and protective immune reactions against targeted pathogens. Consequently, this approach has substantially reduced the time required for developing viable vaccine candidates compared to traditional methods. AI has revolutionized the field of drug discovery by enabling researchers to accurately predict drug–protein interactions and efficiently design lead compounds [98]. Furthermore, AI has improved the process of drug development by automating various tasks and reducing the cost and time associated with preclinical and clinical trials.

#### 4.7.3. Epidemiological Forecasting

AI-powered epidemiological models incorporate intricate data streams for predicting the transmission of the virus. These models analyze factors such as infection rates, hospitalization rates, population density, and human mobility. Machine learning algorithms play a role in refining these models by adapting them to real-world data. By simulating different scenarios based on varying intervention strategies, these models offer policymakers valuable insights into the potential consequences of their actions. Consequently, they aid in resource allocation decisions, inform the implementation of public health measures, and help to mitigate stress on healthcare systems during periods of high demand. AI-powered epidemiological models have become invaluable tools in predicting the transmission of viruses, including COVID-19. Furthermore, AI-based techniques can be utilized to track and monitor the spread of viruses at different scales, ranging from individual to population levels [99].

#### 4.7.4. Remote Monitoring and Telehealth

AI-enabled remote monitoring tools have emerged as a valuable solution for facilitating patient care from the comfort of home. Through the use of wearable devices, vital signs and symptoms can be continuously tracked to generate streams of data. These extensive datasets are then subjected to AI algorithms that accurately identify any deviations from baseline levels, allowing healthcare providers to promptly address potential declines in patients’ conditions. Additionally, telehealth platforms employ sophisticated chatbots driven by AI capabilities that aid patients in conducting symptom assessments, providing them with relevant information and determining the urgency required for medical attention. By leveraging these advanced systems, healthcare professionals can effectively enhance patient care while simultaneously mitigating unnecessary face-to-face interactions, an especially crucial measure during times such as widespread pandemics [100].

#### 4.7.5. Data Analysis and Decision Support

The COVID-19 pandemic resulted in a significant influx of data, ranging from the number of cases to hospitalizations to genomic sequences. AI systems have played a crucial role in analyzing these datasets to uncover patterns that contribute to decision-making processes. Machine learning algorithms specifically help to identify risk factors correlated with severe outcomes and demographic trends regarding infections and to evaluate the effectiveness of interventions. The insights derived from these analyses can assist public health officials and policymakers in making well-informed choices, effectively allocate resources, and tailor interventions to cater to specific populations [101].

#### 4.7.6. Natural Language Processing

Natural language processing techniques facilitate the comprehension and interpretation of human language by AI. In light of the COVID-19 pandemic, NLP tools empowered by AI are capable of systematically sifting through an expansive repertoire of scientific literature to extract significant insights. By summarizing research discoveries and identifying emerging patterns, these advanced tools assist researchers in promptly accessing pertinent information [102]. Furthermore, NLP drives the development of chatbots that deliver precise information to the general public, effectively addressing concerns and countering false or misleading narratives [103].

#### 4.7.7. Contact Tracing

AI-powered contact tracing applications utilize smartphone technologies such as Bluetooth to monitor and record interactions among individuals. Advanced algorithms powered by machine learning assess the likelihood of disease transmission by considering factors such as proximity, duration, and contextual information [104]. To uphold privacy standards, these apps employ decentralized data storage and cryptographic techniques that safeguard sensitive data from unauthorized access. Through the implementation of AI technology, public health agencies can effectively trace potential infection routes, control outbreaks, and promptly notify individuals who may be at risk [105]. However, it is important to address privacy concerns that arise with the use of AI-powered contact tracing applications. One way to address privacy concerns in AI-powered contact tracing applications is by incorporating privacy-by-design principles [106]. Privacy-by-design principles ensure that privacy is considered at every stage of the design and development process of AI-powered contact tracing applications. By incorporating privacy-by-design principles, contact tracing apps can prioritize user privacy and data protection [107]. Additionally, contact tracing apps can adopt a decentralized approach to data storage. This means that personal data are stored locally on users’ smartphones rather than being collected and stored centrally. This approach enhances privacy, as it minimizes the risk of unauthorized access to sensitive information. Furthermore, adopting an opt-in system for contact tracing apps can help to address privacy concerns. Adopting an opt-in system for contact tracing apps means that users have the choice to voluntarily participate in the app’s functionality. This empowers individuals to make informed decisions about their privacy and control the collection and use of their personal data. Overall, the use of AI-powered contact tracing applications presents significant benefits for public health agencies in controlling disease transmission. However, the collection of personal data and potential surveillance associated with these apps continue to raise valid privacy concerns.

#### 4.7.8. Patient Triage and Resource Allocation

AI-driven triage systems utilize advanced algorithms to assess patient information such as medical history, symptoms, and laboratory findings in order to anticipate the probability of severe illness [108]. These machine learning models detect early signs of complications, allowing healthcare professionals to prioritize individuals requiring urgent care. In situations where resources are scarce, AI technologies facilitate the efficient allocation of resources by considering predicted patient outcomes; this ensures optimal utilization of critical supplies like ventilators. In addition to patient triage, AI-based systems play a crucial role in resource allocation within hospitals and on a larger scale. By accurately predicting disease severity and patient outcomes, AI-driven triage systems aid in clinical decision-making as well as in the planning and allocation of resources across hospital systems and at the state/country level [109]. Moreover, the use of AI in patient triage and resource allocation becomes particularly vital during pandemics. During the COVID-19 pandemic, AI algorithms demonstrated their effectiveness in predicting patient outcomes and identifying individuals at high risk for severe illness. These AI-powered systems consider a wide range of factors, including pre-existing conditions, laboratory results, and in-hospital data.

### 4.8. Medical Training

The effectiveness of medical education is closely linked to a doctor’s ability to provide proper care for their patients while minimizing the risk of irreversible errors or harm. As a result, physicians undergo extensive training over several years to acquire a well-rounded set of skills encompassing cognitive, psychomotor, and affective domains. In the past, medical education primarily emphasized the retention of large volumes of complex information for long periods. However, this task has become increasingly challenging in light of the vast amount of new knowledge being generated daily, which surpasses human capacity [110]. The rapid advancement of AI in the medical field has demonstrated the ability to effectively store, analyze, and retrieve medical data, saving clinicians valuable time and energy in patient management and treatment. This integration of cutting-edge technology necessitates a reformation in the education of future healthcare providers in order to align with these recent advancements, particularly the incorporation of AI into medical practice.

One benefit of incorporating AI into medical education is its ability to enhance the efficiency of learning and studying. This in turn reduces the workload for students and provides them with more opportunities to improve their fundamental clinical skills [111]. In an increasingly demanding healthcare industry, it is crucial for medical professionals to have proficient information recall and analysis abilities as well as to possess effective communication skills, manual dexterity in performing clinical tasks, cultural sensitivity, and empathy toward patients. The integration of AI technologies offers a valuable educational resource that streamlines the didactic components of medical training. Consequently, this allows students to prioritize developing their clinical expertise and interpersonal competencies while promoting a compassionate approach within the field of medicine [112]. Additionally, AI in medical education can facilitate the acquisition of practical skills through real-world use of technologies [113]. This can be achieved by incorporating AI-based tools such as intelligent tutoring systems that simulate interactive scenarios for students to practice their clinical decision-making and problem-solving skills

The integration of AI in medical training offers the potential for a more personalized educational experience tailored to meet the individual needs of students, thereby enhancing their overall performance [114]. The utilization of AI has proven effective in analyzing students’ progress and identifying specific areas where knowledge gaps exist. This enables immediate and customized feedback, empowering students to learn at a pace that suits their abilities and preferences [114]. Additionally, AI-powered tools can provide valuable resources for medical education, such as computer-based models, virtual reality simulations, and personalized learning platforms [115]. These resources allow students to experience real-world scenarios in a safe and controlled environment, enabling them to develop practical skills and apply theoretical knowledge.

The incorporation of AI-based virtual reality simulations in medical education can offer significant benefits by providing a safer learning environment for students to refine their clinical skills before entering real-world patient care. This approach allows for practice in common scenarios such as suturing or conducting physical examinations while enabling preparation for rare yet potentially impactful catastrophic events. For example, research has demonstrated the feasibility of simulating Operating Rooms using AI technology, which proved effective in analyzing healthcare professionals’ response to such emergencies and delivering appropriate training on how to handle these unique situations safely and effectively [116]. Furthermore, AI-driven virtual reality simulations have the potential to revolutionize radiology education [117]. With the integration of AI intelligence, radiology simulations in virtual environments can reproduce real-world scenarios and provide learners with immersive, interactive, and realistic training experiences that closely mimic clinical practice.

The incorporation of AI in the field of medicine is yet to be fully realized, resulting in a knowledge and skills gap among clinicians regarding its application in their day-to-day practice. This lack of familiarity with AI hinders clinicians from harnessing its numerous benefits within the medical sector. This is not due to a lack of interest in becoming proficient at utilizing AI on their part; rather, it can be attributed to the limited availability of educational resources for both students and practicing clinicians. A recent experimental study that introduced an AI course specifically designed for fourth year medical students demonstrated promising outcomes, as evidenced by an average score of 97% achieved by the participants [118]. These findings validate the effectiveness of online modules focusing on AI and emphasize the pressing need for additional educational opportunities within current medical training programs. The integration of AI into medical practice requires a thorough understanding of its potential benefits and limitations. This prompts medical educators to teach the best practices of AI as a tool while understanding its limitations [119]. To bridge the knowledge gap and enhance the understanding of AI among medical professionals, medical educators need to develop standardized AI content and incorporate it into medical training pathways [120]. This approach will ensure that medical students and clinicians are equipped with the necessary knowledge and skills to effectively utilize AI in their practice.

## 5. Considerations of AI Applications

The integration of AI in the healthcare sector has the potential to greatly enhance patients’ overall experience with medical care. As previously mentioned, AI can be implemented across various areas within medicine [121]. However, it is important to address certain considerations such as biases and public awareness surrounding AI.

### 5.1. Ethical and Legal Considerations around AI

There are a number of relevant ethical and legal issues that need to be carefully considered moving forward. (a) Accountability and Liability: as AI systems take on increasingly complex tasks, determining responsibility for mistakes becomes a challenge. If an AI-driven diagnosis leads to harmful medical errors, it is uncertain where the liability rests; is it the healthcare provider, the developers of the AI system, or the machine itself? This legal conundrum needs to be addressed with comprehensive regulations addressing accountability and liability in AI applications in healthcare. (b) Privacy Protection: with AI systems processing vast amounts of sensitive patient information, ensuring data privacy becomes paramount. Patient data can be misused or fall into the wrong hands, making it crucial to have strong data protection measures in place. The legal frameworks regarding data privacy in the context of AI should adhere to principles of data minimization, purpose limitation, and secure data processing methods. (c) Bias and Discrimination: AI systems can unintentionally perpetuate existing biases present in healthcare data, leading to unequal treatment or inaccurate diagnoses for certain groups. Case studies on issues such as racial bias present in healthcare AI algorithms underline the importance of anti-bias measures in the development and deployment of these systems. Regulatory bodies must enforce guidelines that ensure fairness and accuracy in the operation of AI systems. (d) Transparency and Explainability: the ‘black-box’ nature of many AI systems can significantly complicate healthcare delivery. When an AI system’s decision-making process is not transparent, healthcare professionals may find it difficult to trust the system’s outputs, affecting implementation. Regulations emphasizing transparency and explainability can facilitate better understanding and acceptance of AI tools in medical practice. (e) Consent: patient consent is another crucial issue when dealing with AI systems. Data used to train AI systems should be collected only after obtaining explicit and informed consent from the patients. Legal requirements need to specify the nature of consent required for different kinds of data usage. (f) Balancing Innovation and Regulatory Oversight: while regulations are necessary to maintain ethical standards, they should not stifle innovation. Policymakers need to ensure that regulatory frameworks provide enough space for the development and application of AI technologies while ensuring patient safety, privacy, and fair treatment.

As the healthcare industry experiences an increased workload resulting from heightened patient demand, digital health technology has garnered more attention as a potential solution. However, this advancement introduces concerns regarding patient privacy and the monitoring of sensitive information. Furthermore, due to the current absence of comprehensive regulations in place, AI systems raise legal and ethical issues such as liability attribution and privacy protection. The challenge lies in identifying responsibility when machines are capable of operating under non-rigid rules and independently acquiring new behavioral patterns [122]. This ambiguity poses a legal conundrum, and accountability for these actions remains uncertain. From an ethical standpoint, safeguarding an individual’s privacy is closely intertwined with their right to autonomy and personal identity within the medical context. Consequently, maintaining strict confidentiality measures while ensuring robust security protocols for AI-based data management is imperative in order to minimize potential breaches or violations of patient privacy. Moreover, the integration of AI in healthcare raises concerns about algorithmic bias and the potential for discriminatory practices [123].

Another issue that may arise is the collection of patient data without informed consent. An example of this can be seen with AI devices gathering and transmitting data from older adults in their homes without their knowledge; these services may provide the collected data to AI developers without patients’ consent [124]. While this has the potential to benefit healthcare through AI implementation, it raises concerns about breaching patient privacy and eroding healthcare trust. Therefore, as mentioned earlier, it is crucial to establish guidelines that set boundaries for how AI utilizes patient data while allowing flexibility for unforeseen circumstances. Integrating ethical and accountable AI solutions into organizational planning is crucial for responsible AI [125]. This ensures the maintenance of trust, minimizes privacy invasion, and meets stakeholder expectations and regulations. Although in its early stages, responsible AI aims to strike a balance between patient needs and long-term economic value in healthcare [126,127]. While leading researchers primarily focus on quantitative evaluation metrics, physicians prioritize trustworthiness, ethics, and providing meaningful explanations for decisions made by deep networks according to data protection laws [128].

The American Medical Association (AMA) has released seven principles for the use of AI in healthcare [129]. The aim is to create a uniform governance framework for the advancement of AI in the industry. The principles stress the importance of comprehensive policies to minimize potential risks associated with AI technologies for patients and physicians. According to the AMA, these principles will aid in optimizing the benefits of AI in healthcare and reducing potential harms. Key aspects within the AMA principles include favoring comprehensive governance for risk management in healthcare AI and promoting transparency with legal requirements in AI design, development, and deployment. In addition, the principles call for privacy-centric AI design, secure handling of personal data, clear disclosure around when AI impacts patient care, and development of policies to address potential negative impacts before deploying generative AI. Furthermore, the AMA urges early identification and mitigation of bias in AI algorithms and advocates for limiting physician liability in the use of AI-enabled technologies while aligning with current medical liability legal frameworks. In the following section, bias and transparency in AI are assessed in more detail.

### 5.2. Bias and Transparency of AI

Often, AI algorithms are developed and trained using healthcare datasets. When these datasets contain inherent biases, the resulting AI system can replicate or even magnify existing disparities. For example, if a dataset used to train a disease prediction algorithm predominantly contains data from certain demographic groups, the algorithm might perform poorly when used on other demographics. This could lead to unequal treatment or misdiagnosis. Bias in decision making is another concern. AI algorithms often replicate the decision-making processes of the health professionals who trained them. If those professionals carry inherent biases, the AI systems might reflect these same biases. As an instance, a study by Obermeyer et al. found that a healthcare algorithm showed racial bias because it was designed to predict healthcare costs rather than sickness [130]. This bias manifested as lower referral rates for black people compared to other racial groups with similar health conditions. To ensure more equitable AI development, efforts must be made to use diverse datasets that accurately represent all population groups. Datasets must be interrogated for potential bias prior to training the algorithms. Furthermore, AI systems should be regularly validated on multiple datasets to ensure that they maintain high performance across diverse populations. AI’s capacity in healthcare depends largely on how the ethical issue of algorithmic bias is addressed. Multidisciplinary efforts involving clinicians, data scientists, ethicists, and policy makers should be enforced to ensure the development and use of equitable and unbiased AI systems in healthcare. AI is meant to improve healthcare accessibility and inequality, not to inadvertently exacerbate existing disparities.

Certain diseases have a higher prevalence in specific genders, which presents a challenge when implementing AI for accurate solutions tailored to certain groups. Gender bias represents one of the obstacles encountered during the deployment of AI systems in medical imaging. For instance, a study by Larrazabal et al. revealed that an imbalanced dataset with unequal representation of males and females led to significantly lower performance averages across all diseases within the minority group, suggesting the presence of gender bias [131]. This highlights the need to consider gender bias when providing data for AI algorithms and to incorporate additional mechanisms that can effectively account for this bias to ensure precise outputs. It is important to address and mitigate bias in AI algorithms, as it can have negative impacts on health, particularly in under-resourced or racial/ethnic minority populations [132].

Obermeyer et al. investigated the potential racial bias present in healthcare AI algorithms used for managing the care of high-risk patients in the United States [130]. These algorithms are responsible for setting up special care management programs mandated by the Affordable Care Act designed to provide extra resources to patients with complex medical needs with the aim of preventing costly medical emergencies. Despite race not being an explicit input into the algorithm, the research found significant racial bias in the algorithm’s predictions. Black patients were substantially less likely to be selected by the algorithm as needing extra care compared to their White counterparts, even when they were just as sick. This disparity mainly stemmed from the algorithm’s focus on health care costs as a proxy for health needs and complexity, which overlooked the fact that less money is spent on Black patients who have the same level of need. The consequent under-identification of high-risk Black patients for care programs has significant implications on the equitable distribution of healthcare resources. These findings suggest the need for careful scrutiny in the design and implementation of healthcare algorithms to ensure that they do not inadvertently propagate systemic biases.

The use of AI in diagnosing various diseases is gaining popularity across multiple medical specialties. AI has demonstrated high accuracy in the diagnosis of dermatological skin lesions. A study conducted at Stanford University in 2017 trained a convolutional neural network using a dataset of over 100,000 images and 2000 diseases. The results showed that the CNN had comparable performance to that of 21 board-certified dermatologists when it came to classifying different types of skin cancer [133]. A study on patient inclinations towards the utilization of AI for identifying skin cancer pathways documented a positive attitude regarding the potential application of AI for this objective [134]. However, it is important to recognize that bias related to skin tone could impact the use of AI in this context. Inadequate representation of varying skin tones in training AI/ML models can lead to erroneous interpretations, especially for marginalized communities. A recent review conducted in 2021 on the use of AI in dermatology examined 70 studies and found that only 10% included skin tone as part of the dataset. Furthermore, it was revealed that darker skin tones were underrepresented in these studies [135]. This highlights a potential flaw when using datasets for diagnosing skin conditions, and emphasizes the need to address this issue. Nonetheless, despite this limitation, incorporating AI into the field of dermatological disorders has significant potential to provide accurate diagnoses and effective treatments.

The transparency of AI has experienced significant growth in recent years. This rapid advancement holds promise for enhancing public awareness and understanding of AI’s applications in healthcare. Addressing the need for clarity on various aspects of AI can foster trust on the part of the public around the integration of AI into medical practice. An important factor in establishing this trust lies in developing a comprehensive understanding of how AI systems interpret information. Lack of knowledge about the inner workings and decision-making processes of these systems creates a gap, particularly among healthcare professionals who may struggle to comprehend their operations within the context of patient care. The difficulty of implementing AI in clinical settings and the clinical translation gap of AI-based tools are well described, and are driven in part by a lack of education and knowledge among clinicians [136]. Improving education and understanding regarding AI applications on the part of healthcare professionals could effectively address the current knowledge gap [137]. This educational initiative would aid in legislation, verification, and system improvement while fostering trust between the individuals involved [138]. It is crucial to focus on comprehending how AI-generated results are interpreted as well as on addressing legal and ethical considerations in order to bolster public confidence in healthcare AI. Furthermore, incorporating cultural constructs and values within the clinical environment is another critical obstacle to overcome in implementing AI in healthcare.

### 5.3. Cybersecurity

The implementation of EHR systems in hospitals worldwide has led to an increased need for cybersecurity measures to ensure patient safety and protect confidential information. There are two crucial reasons why cybersecurity is imperative in modern healthcare: the digital storage of sensitive patient data, and the vulnerability of electronic systems to remote access by hackers due to inadequate defenses [139]. Instances of cybercrime within the healthcare sector include ransom attacks, theft of patient health data, and unauthorized control over implanted medical devices, all of which pose serious risks to individuals’ well-being. Additionally, cybersecurity plays a critical role in maintaining privacy and trust on the part of patients [140]. One main issue with cybersecurity in healthcare is the abundance of valuable data, including personal and medical information. The other issue is the weak defenses that are typically found in healthcare systems, making them easy targets for cyberattacks. The reliance on electronic health records and other core systems in healthcare means that an effective cybersecurity framework is necessary to protect these systems from threats [141]. In contrast to physical breaches, which involve accessing patient records directly, a single medical device can serve as an entry point into the health record software of any hospital [142]. The use of AI introduces new opportunities for attackers to compromise patient privacy and undermine healthcare security, which is already vulnerable; therefore, it is crucial to develop legislation that protects patient information and to implement effective digital safeguards before the widespread adoption of AI algorithms in medicine [143]. Fortunately, there is potential for AI itself to contribute to maintaining patient privacy through computational text de-identification algorithms, which may offer a faster and more cost-effective solution compared to human experts [144].

### 5.4. Public Opinion around AI in Healthcare

**Figure 5:**
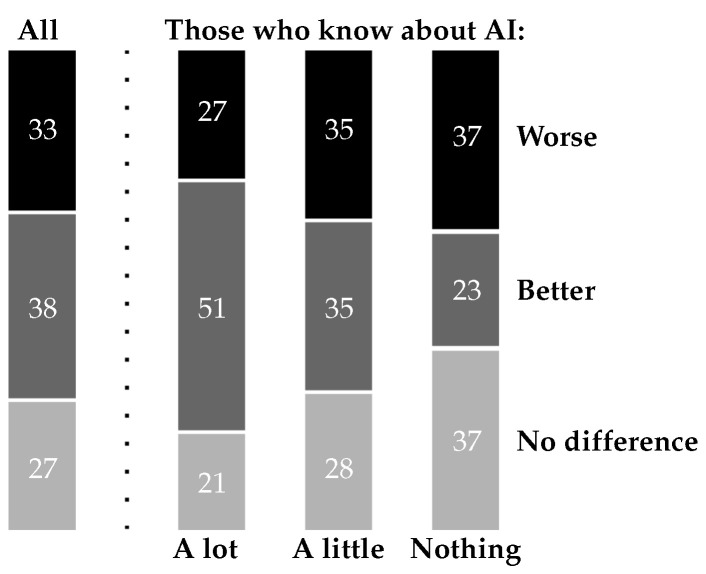
Public opinion on AI and whether it will lead to better/worse/similar outcomes for patients. (Source: Pew Research Center [146]). Note: Respondents who did not give an answer are not shown.

The implementation of AI in healthcare is progressing rapidly, and it is crucial to consider public opinion on its integration. While there are vast possibilities for AI to enhance the healthcare sector, it is important to acknowledge that public perception can significantly impact how AI technologies are adopted. This includes the public perspective on various aspects, such as data collection during early stages of medical care, as well as acceptance and trust in using AI for diagnoses and treatments later on [145]. It is clear that the public’s perception of AI in healthcare is not homogeneous and that there are varying levels of acceptance and apprehension. Recent surveys conducted by Pew Research have assessed public sentiment across multiple categories, including reliance on and impact of AI in medical care, knowledge about diagnosis and treatment methods, utilization of surgical procedures and pain management techniques, and the use of chatbots for mental health support [146]. In general, the majority of individuals express discomfort with providers relying solely on AI for medical care. There is a divide in opinion regarding whether this reliance improves, worsens, or has no significant impact on overall healthcare outcomes. The public’s perception of AI in healthcare is influenced by factors such as education, knowledge about AI, and previous experiences [147]. Similarly, the findings presented in Figure 5 indicate that individuals with higher education and knowledge about AI tend to have greater confidence in its capacity to enhance patient outcomes within the healthcare sector. Hence, individuals with higher education tend to perceive AI as a valuable tool in enhancing patient care, facilitating early detection and diagnosis, optimizing treatment plans, and improving overall healthcare outcomes. However, according to the survey findings the majority of respondents expressed reservations about using AI for determining pain medication after surgery or relying on surgical robots for procedures. Furthermore, most participants believed that incorporating AI into pain management would either exacerbate their symptoms or yield no significant differences. Similarly, a significant majority indicated an unwillingness to utilize an AI chatbot for mental health support unless they were concurrently seeing a therapist [148]. These opinions held true both among those familiar with chatbots and those who had no previous knowledge of them. Overall, this survey found that while there is an increasing awareness and interest in AI’s potential in healthcare, there was also skepticism and concern among the public. This skepticism and concern may stem from a lack of understanding or familiarity with AI as a concept as well as apprehension about data privacy and control [146]. To address this, it is important to conduct comprehensive research that takes into account the public’s perspective from various qualitative and quantitative studies, even though public perceptions of AI in healthcare are not limited to concerns about trust and reliance on technology.

The survey findings indicate a strong association between knowledge of AI and belief in its potential to have a positive impact on healthcare. Participants who demonstrated a higher level of understanding were more inclined to embrace the integration of AI into their overall medical treatment. However, this correlation weakened when it came to specific areas such as mental health, where individuals may have been less receptive towards incorporating AI into their treatment for these conditions. Nevertheless, AI has diverse applications in mental healthcare beyond the scope of chatbots alone. While patients may show decreased interest in using AI for treating their mental health issues, it can offer benefits for diagnostic purposes. For example, AI-based speech analysis techniques have been developed to identify depression [149] and wearable AI devices have the potential to detect anxiety, although their current level of advancement does not yet allow for clinical use [150].

The surveys reveal an overarching lack of awareness among the general public regarding AI in healthcare settings, emphasizing the necessity for targeted efforts aimed at educating and informing individuals about its capabilities within this domain. Addressing this knowledge gap is crucial for fostering effective collaboration between AI initiatives and healthcare practices in order to enhance patient care outcomes and optimize the use of resources in the healthcare system. Additionally, it can be inferred that healthcare specialists who have a deeper understanding of AI are more inclined to embrace and utilize this technology in their professional roles to enhance patient care.

While the general sentiment towards the utilization of AI in healthcare remains predominantly positive, it is crucial to acknowledge and address certain prevailing challenges that demand increased attention. Merely focusing on algorithmic concerns is insufficient; it is imperative to delve deeper into the practical implementation of legislation and guidelines to ensure the incorporation of principles such as fairness, accountability, transparency, and ethics within AI systems deployed in healthcare settings [151]. This comprehensive approach will contribute to safeguarding the integrity and responsible use of AI technology in healthcare.

## 6. Discussion

As the digital revolution continues to accelerate, it is clear that AI is becoming increasingly integral to healthcare. Its benefits, which include enhancing diagnostic accuracy, personalizing treatments, and increasing operational efficiency, have the potential to profoundly improve patient outcomes and reduce the burden on healthcare systems around the world. However, it is important to acknowledge that successful integration of AI into healthcare practices demands more than just technical proficiency. The ethical and social implications of AI usage in healthcare present considerable challenges. There is an urgent need to address issues such as data privacy, algorithmic bias, and accountability. Ensuring fairness, reliability, and transparency should be a priority in AI development. Moreover, collaboration between AI specialists, healthcare professionals, policy makers, and patients is key to developing AI tools that can truly meet the needs of all stakeholders. Additionally, due to the complex nature of medical decision-making, the role of AI should be envisioned as a support tool for clinicians rather than a replacement for human judgment. The human element in healthcare, which encompasses empathy, complex reasoning, and understanding of individual patient needs, cannot be replicated by an algorithm. Lastly, it is necessary to shift focus towards education and public awareness about AI in healthcare. Encouraging public understanding and dialogue about AI will be crucial for building trust and acceptance in the system. With a balanced and thoughtful approach, AI has the capacity to transform healthcare, bringing enormous benefits to both patients and providers. The rapidly evolving field of AI in healthcare will continue to offer exciting opportunities for further exploration and discovery in future work.

The field of AI has experienced rapid advancements and is now being utilized across various industries. Originally designed as a large computer for deciphering war signals, AI has evolved into a sophisticated cloud-based neural network. It functions as an adaptable tool that enhances complex human tasks in different sectors. In the medical domain specifically, the implementation of AI shows immense potential for future applications [152]. By demonstrating proficiency in surgical procedures, administrative responsibilities, diagnostics, imaging techniques, medical education, and patient management, AI can significantly alleviate healthcare burdens while fostering greater efficiency. This collaborative approach ensures that AI algorithms work alongside clinicians to enhance health outcomes and quality of care without posing any threat to job security or replacing human expertise in the healthcare field.

Collaboration among clinicians, educators, researchers, and developers is essential for the successful integration of AI into healthcare in the coming decade. Despite the impressive advancements made by AI, there remain many important considerations that need to be addressed. These include addressing biases and ensuring transparency as well as tackling legal and ethical challenges. Additionally, it is imperative to take into account public opinion and awareness regarding the incorporation of AI in medicine, as this will greatly influence progress within the field. Nonetheless, despite the need for these challenges to be resolved over time, AI has already demonstrated numerous promising capabilities that have tremendous potential for improving healthcare delivery and shaping the future of medicine. It should be emphasized that while AI shows immense promise, it necessitates rigorous validation processes along with ongoing collaboration between experts in AI and medical professionals to ensure safe implementation practices yielding effective results.

In light of the aforementioned challenges associated with AI in healthcare, the AMA has introduced guidelines pertaining to the advancement and application of AI in the healthcare sector. These guidelines aim to tackle critical concerns, including data privacy, cybersecurity, and the utilization of AI by insurance providers [129]. The report emphasizes the “quadruple aim” for AI systems: to enhance patient care and outcomes, improve population health, reduce healthcare costs while increasing value, and support the wellbeing of healthcare professionals. The guidelines further stress the need for regulatory oversight, transparency, data protection, and the avoidance of exacerbating inequities in healthcare while aiming to ensure AI tools meet both physician and patient needs.

## 7. Conclusions

An essential point to underscore is the collaborative potential between AI and healthcare professionals. AI is best viewed not as a replacement for healthcare professionals but as an adjunct tool, that is, a partner that can handle extensive data analysis, provide diagnostic support, and free up more time for healthcare professionals to spend on direct patient care. The successful implementation of AI would enable healthcare providers to leverage their unique skills of empathy, complex decision-making, and direct patient communication that AI cannot emulate. Rather than eliminating jobs, AI is more likely to transform them, shifting healthcare professionals’ emphasis to duties that complement AI technologies. Nevertheless, it is worth noting that along with the exciting advancements and numerous advantages there are challenges that need to be tackled. Key among these are the legal and ethical complexities inherent in AI’s integration into healthcare. Moving forward, there is significant potential for AI in healthcare; however, realizing this potential will require continued research, cross-disciplinary cooperation, and open dialogue among AI developers, healthcare professionals, and ethical and legal experts. Combining AI’s capabilities with the human elements of healthcare professionals will lead to more personalized, effective, and efficient patient care.

## Figures and Tables

**Table 1:** Examples of AI healthcare companies in the US.

Name	Location	Year	Operation
Accuray	Sunnyvale, CA	1990	Robot-assisted surgeries.
Intuitive	San Francisco, CA	1995	Robot-assisted surgeries.
Auris Health	Redwood City, CA	2007	Robot-assisted surgeries.
Qventus	Mountain View, CA	2011	Management of in-patient & outpatient flow.
H2O.AI	Mountain View, CA	2011	Improves clinical workflow, analyzes patient risk & predicts ICU transfers.
Atomwise	San Francisco, CA	2012	Drug development.
Enltitic	San Francisco, CA	2014	Improves quality & efficiency of medical imaging to diagnose & treat diseases.
Freenome	San Francisco, CA	2014	Analyzes blood tests to Identify & treat the earliest stages of life-threatening cancers.
Buoy Health	Boston, MA	2014	Listens to patient’s symptoms & gives guide for proper treatment.
CloudMedX	San Francisco, CA	2015	Tracks & manages the patient’s journey across the healthcare system.
Tempus	Chicago, IL	2015	Collects & processes clinical and molecular data of patients.
KenSci	Seattle, WA	2015	Detects clinical & financial risks in healthcare organizations.
PathAI	Boston, MA & Austin, TX	2016	Fast diagnosis/treatment of pathologic diseases.

**Table 2:** Ten applications of AI in healthcare with transformative potential (Source: Accenture; hbr.org, accessed on 4 January 2024).

Application	Potential Annual Value	Assists with…
Robot-assisted surgery	$40B 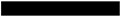	more types of surgery.
Virtual nursing assistant	$20B 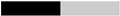	medical labor shortage.
Administrative workflow	$18B 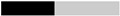	integration with existing infrastructure.
Fraud detection	$17B 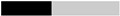	complex fraud attempts.
Dosage error reduction	$16B 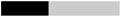	medical errors.
Connected machines	$14B 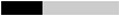	proliferation of connected devices.
Clinical trial participation	$13B 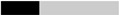	plethora of data.
Preliminary diagnosis	$5B 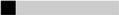	enhancing diagnosis accuracy.
Image diagnosis	$3B 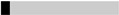	storage capacity.
Cybersecurity	$2B 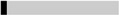	protecting health data.

## Data Availability

Not applicable.

## Abbreviations

The following abbreviations are used in this manuscript:

AI	Artificial Intelligence
CT	Computer Tomography
ECG	Electrocardiogram
CAD	Computer-Aided Diagnosis
MRI	Magnetic Resonance Imaging
EUS	Endoscopic Ultrasound
ICU	Intensive Care Unit
EHR	Electronic Health Record
ANN	Artificial Neural Network
DL	Deep Learning
DLN	Deep Learning Network
CNN	Convolutional Neural Network
NLP	Natural Language Processing
ML	Machine Learning
US	United States
USD	United States Dollar
HF	Heart Failure
DR	Diabetic Retinopathy
STAR	Smart Tissue Autonomous Robot
COVID-19	Coronavirus Disease 2019
SARS	Severe Acute Respiratory Syndrome
AMA	American Medical Association
